# A WAO - ARIA - GA²LEN consensus document on molecular-based allergy diagnostics

**DOI:** 10.1186/1939-4551-6-17

**Published:** 2013-10-03

**Authors:** Giorgio Walter Canonica, Ignacio J Ansotegui, Ruby Pawankar, Peter Schmid-Grendelmeier, Marianne van Hage, Carlos E Baena-Cagnani, Giovanni Melioli, Carlos Nunes, Giovanni Passalacqua, Lanny Rosenwasser, Hugh Sampson, Joaquin Sastre, Jean Bousquet, Torsten Zuberbier

**Affiliations:** 1 Allergy & Respiratory, DIMI, Department of Internal Medicine, University of Genoa, Largo Rosanna Benzi, Genoa, Italy; 2 Department of Allergy and Immunology, Hospital Quirón Bizkaia, Carretera Leioa-Inbe, Erandio (Bilbao), Spain; 3 Department of Pediatrics, Division of Allergy, Nippon Medical School, Tokyo, Japan; 4 Department of Dermatology, Allergy Unit, University Hospital of Zurich, Zurich, Switzerland; 5 Clinical Immunology and Allergy Unit, Department of Medicine Solna, Karolinska Institutet and University Hospital, Stockholm, Sweden; 6 Research Centre for Respiratory Medicine, Catholic University, Cordoba, Argentina; 7 Dipartimento di Medicina Sperimentale, Istituto Giannina Gaslini, Genova, Italy; 8 Centre of Allergy of Algarve, Algarve, Portugal; 9 University of Genoa, Genoa, Italy; 10 University of Missouri – Kansas City School of Medicine, Children’s Mercy Hospital and Clinics, Kansas, KS, USA; 11 Department of Pediatrics, Division of Allergy/Immunology, Icahn School of Medicine at Mount Sinai, New York, NY, USA; 12 Department of Medicine, Fundacion Jimenez Diaz, Universidad Autonoma de Madrid, Avenida Reyes Catolicos, Madrid, Spain; 13 Service Maladies Respiratoires, Hopital Arnaud de Villeneuve, Av. Doyen Gaston Giraud, Montepellier, France; 14 Clinic for Dermatology and Allergy Charité Universitätsmedizin Berlin, Charitéplatz 1, 10117 Berlin, Germany

## Abstract

Molecular-based allergy (MA) diagnostics is an approach used to map the allergen sensitization of a patient at a molecular level, using purified natural or recombinant allergenic molecules (allergen components) instead of allergen extracts. Since its introduction, MA diagnostics has increasingly entered routine care, with currently more than 130 allergenic molecules commercially available for *in vitro* specific IgE (sIgE) testing.

MA diagnostics allows for an increased accuracy in allergy diagnosis and prognosis and plays an important role in three key aspects of allergy diagnosis: (1) resolving genuine versus cross-reactive sensitization in poly-sensitized patients, thereby improving the understanding of triggering allergens; (2) assessing, in selected cases, the risk of severe, systemic versus mild, local reactions in food allergy, thereby reducing unnecessary anxiety for the patient and the need for food challenge testing; and (3) identifying patients and triggering allergens for specific immunotherapy (SIT).

Singleplex and multiplex measurement platforms are available for MA diagnostics. The Immuno-Solid phase Allergen Chip (ISAC) is the most comprehensive platform currently available, which involves a biochip technology to measure sIgE antibodies against more than one hundred allergenic molecules in a single assay. As the field of MA diagnostics advances, future work needs to focus on large-scale, population-based studies involving practical applications, elucidation and expansion of additional allergenic molecules, and support for appropriate test interpretation. With the rapidly expanding evidence-base for MA diagnosis, there is a need for allergists to keep abreast of the latest information. The aim of this consensus document is to provide a practical guide for the indications, determination, and interpretation of MA diagnostics for clinicians trained in allergology.

## Introduction

•***In summary, ****molecular allergy (MA) diagnostics is increasingly entering routine care, and currently, more than 130 allergenic molecules are commercially available for in vitro specific immunoglobulin E (sIgE) testing.*

•*MA diagnostics may initially appear complicated; however, with increasing experience, the information gained is generally simple and provides relevant information for the allergist. This is especially true with regard to food allergy and for the selection of specific immunotherapy.*

•*Nevertheless, all sIgE tests including MA diagnostics should be evaluated within the framework of a patient’s clinical history, since allergen sensitization does not necessarily imply clinical responsiveness.*

•*Clinicians and immunologists specifically trained in allergology must keep abreast of the new and rapidly evolving evidence available for MA diagnostics.*

In the late 1960s, the discovery of the immunoglobulin (IgE) antibody provided a specific biomarker that could be used to identify allergic diseases triggered by environmental allergens (i.e., generally proteins). Traditional IgE antibody tests such as skin prick tests (SPT) or *in vitro* specific IgE (sIgE) tests are based on crude extracts composed of allergenic and non-allergenic molecules obtained from an allergenic source. With the application of DNA technology in the late 1980’s, allergenic molecules were characterized and cloned in order to resolve the determinants of various allergic diseases [1-4]. The availability of allergenic molecules in the last decade has ushered in a new phase of diagnostics, termed molecular-based allergy (MA) diagnostics, that allows for improved management of allergic diseases [5].

Today, many of the most common allergenic molecules have been cloned or purified, have had their three-dimensional structures elucidated, and can be consistently produced [6]. Because of the growing number of allergens identified, a systematic allergen nomenclature, approved by the World Health Organization and International Union of Immunological Species (WHO/IUIS) Allergen Nomenclature Subcommittee, has been established. The subcommittee is in charge of developing and maintaining the systematic nomenclature developed for allergenic molecules, as well as a comprehensive database of known allergenic proteins that can be accessed at http://www.allergen.org. Allergenic molecules are named using their Latin family name (genus and species). For example, allergens that begin with **Phl p** are from ***Phl****eum ****p****ratense* (timothy grass). A number is added to the name to distinguish the various allergens from the same species (e.g., Phl p 1, Phl p 2, etc.). The numbers are assigned to the allergens in the order of their identification. Allergenic molecules are classified into protein families, according to their structure and biological function [7]. Many different molecules share common epitopes (antibody binding sites) and the same IgE antibody can bind and induce an immune response to allergenic molecules with similar structures from various allergen sources. These cross-reactive allergens give valuable information regarding sensitization to several different sources. In contrast, some molecules are unique markers for specific allergen sources, allowing for the identification of the primary sensitizer.

MA diagnostics is increasingly entering routine care and can improve management of allergic patients. This is particularly evident in food allergy [8-10]. Knowledge of the allergenic molecules the patient is sensitized to can help to discriminate between likelihood of local versus systemic reactions and persistence of clinical symptoms. For example, some allergens such as storage proteins in peanuts (e.g. Ara h 2) and nuts (e.g. Cor a 9) have been shown to be associated with severe reactions, while other allergens cause sensitization mostly without a clinical reaction. Another important aspect, difficult to elucidate using traditional tests, is the stability of the allergen. Allergens that are stable to heat and digestion (e.g., Ara h 2 from peanut) are more likely to cause severe clinical reactions, whereas heat and digestion labile molecules (e.g., Ara h 8 from peanut) are more likely to cause milder, local reactions or be tolerated. Similarly, identifying whether the sensitization is genuine in nature or due to cross-reactivity help to evaluate the likelihood of reaction on exposure to different allergen sources [8]. Molecular diagnostics may also improve the selection of both patients and specific allergens for specific immunotherapy (SIT) for inhalant allergies (e.g., for pollen) [11,12] and hymenoptera venom allergy [13,14]. An ever increasing number of studies focusing on different allergenic molecules or allergic diseases are rapidly being published. However, the search for more, clinically relevant molecules is needed and ongoing.

The presence of IgE antibodies against allergenic molecules may be determined using a singleplex (one assay per sample) or multiplex (multiple assays per sample) measurement platform. A singleplex platform allows the doctor to select those allergenic molecules necessary for an accurate diagnosis defined by the clinical history of the patient. The multiplex approach allows for characterization of the IgE response against a broad array of pre-selected allergens on a chip independently of the clinical history. There is one commercially available multiplex immuno-solid phase allergen chip (ISAC) which contains more than 100 allergens from about 50 allergen sources. The large number of allergens provides extensive and detailed information about a patient’s sensitization profile [12,15]. ISAC is especially suited for use in patients with complex sensitization pattern or symptoms. The ISAC technology is a promising MA approach for improved diagnosis, prognosis, and selection of patients for SIT. While it is a commercial product, it has been the mainstay of many investigator studies so far.

In summary, with increasing experience, MA diagnostics is generally straightforward to interpret and can provide relevant, additional information for the allergist. However, the clinical utility of many of the allergenic molecules needs further investigation. Because of the speed at which new data on MA diagnostics is becoming available, clinicians are required to keep pace with a large amount of novel information. This WAO - ARIA - GA^2^LEN consensus document on Molecular-based allergy diagnostics provides a practical guide for the indication, determination, and interpretation of MA diagnostics that is aimed for clinicians specifically trained in allergology.

## Definitions and concepts

### Allergen source

A tissue, particle, food or organism inducing allergy (e.g. cat dander, *D. pteronyssinus*, milk, *Aspergillus fumigatus*, *Phleum pratense* pollen, etc.).

### Allergen extract

A crude, unfractionated mixture of allergenic and non-allergenic proteins, polysaccharides, and lipids obtained by extraction from an allergen source (e.g., pollen grains).

### Allergenic molecule (allergen component)

A molecule (i.e., protein or glycoprotein) derived from a given allergen source that is identified by sIgE antibodies (hereafter referred to as allergen). Allergens can be isolated from natural allergen sources (native, purified allergen) or can be produced by using recombinant DNA technology (recombinant allergen).

#### Stability of allergens

Allergens that are susceptible to **acid pH** in context with peptic digestion (relevant at the gastric level) are not able to cross the gastric barrier (except possibly in patients treated with antacid drugs [16]). **Temperature** (cooking or boiling) susceptibility indicates that the allergen does not maintain its allergenicity after cooking/heating procedures. Heating may occur in the industrial processing of food for production and in domestic cooking. The structure of allergens susceptible to **protease digestion** is affected by gastric and pancreatic enzymes. Accordingly, allergens sensitive to these factors are considered labile, while those that are not are considered to be stable.

#### More about allergenic molecules

A **genuine** allergen causes specific sensitization to its corresponding allergen source. **Major** allergens are defined as those that bind to IgE in 50% or more of patients with the same allergy; in other words, the majority of patients (≥50%) with the same allergy are sensitized to the allergen in question. A **primary** allergen is the original sensitizing molecule (i.e., the driving trigger; in contrast to secondary sensitization due to cross-reactivity). In general, major allergens are also genuine and primary. Finally, the **abundance** of a molecule present in the allergen source is also a parameter to take into consideration.

**Cross-reactivity:** the phenomenon of an IgE antibody recognizing, binding, and inducing an immune response to similar allergenic molecules (homologues) present in different species; for example, an IgE antibody that binds and reacts to both Bet v 1 in birch pollen and Cor a 1 in hazelnut due to their structural similarity (generally characterized by greater than 50%-70% sequence homology between the primary structures of the proteins). IgE cross-reactivity often occurs between the following:

a) Allergenic molecules in closely related species (e.g., between grass or between mite allergens);

b) Well preserved molecules with similar function present in widely different species that belong to the same protein family (e.g., members of the tropomyosin protein family, such as Der p 10 in house dust mite and Pen m 1 in black tiger shrimp).

### Component resolved diagnostics (CRD)

See molecular-based allergy diagnostics.

### Co-sensitization

Genuine sensitization to more than one allergen source (e.g., timothy grass and birch), where the sensitization is not due to cross-reactivity.

### CCD

Cross-reactive carbohydrate determinant. CCDs are carbohydrate moieties of glycoproteins. The most commonly described is MUXF3 [17].

### Epitope

The region of the protein recognized and bound by an antibody (i.e., the antibody binding site).

### Molecular-based allergy (MA) diagnostics

A diagnostic approach to define the allergen sensitization of a patient at the molecular level using purified natural or recombinant allergen on singleplex or multiplex measurement platforms.

### Pan-allergen

A cross-reactive allergen, belonging to a protein family well preserved throughout many widely different species, able to trigger IgE antibody binding (e.g., profilins or serum albumins). See also definition of cross-reactivity (b).

### Recombinant allergen

An allergenic molecule produced using DNA cloning and protein purification techniques. Recombinant allergens can be produced with consistency in terms of quality and amounts, and without CCD structures. Allergen extracts cannot be produced by recombinant techniques.

### sIgE concentration/level

a) High level: represents a high concentration of sIgE antibodies specific for an allergenic extract or molecule. Generally, the higher the sIgE level the higher the probability of clinical reactions. Some allergens also have a high probability of inducing severe reactions at low sIgE concentrations (e.g., storage proteins and lipid transfer proteins [LTPs]), while others typically do not result in any clinical reactions despite high sIgE concentrations (e.g., cross-reactive carbohydrate determinants [CCD]).

b) Low level: Represents a low concentration of sIgE antibodies specific for an allergen extract or molecule.

### sIgE sensitization

Presence of allergen-specific IgE (sIgE) antibodies in the blood that may occur in the presence or absence of clinical symptoms.

a) **Mono-sensitization**: Sensitization to one allergen source (*Dermatophagoides pteronyssinus*) or to a closely related taxonomical family or group of allergen sources (i.e., mites).

b) **Poly- (or multi-) sensitization:** Sensitization to three or more allergen sources (e.g., mite, birch, and grass pollen).

### sIgE detection based on allergen extracts

Singleplex or multiplex platforms for *in vitro* measurement of sIgE reactivity to allergen extracts. Terms such as CAP, radioallergosorbent test (RAST), sIgE and *in vitro*-test are often used interchangeably for this technique. However, the performance of different measurement platforms differs and this should be taken into consideration when reporting and comparing results. This approach cannot identify cross-reacting molecules.

### sIgE detection based on allergenic molecules

Singleplex or multiplex platforms for *in vitro* measurement of sIgE reactivity to allergenic molecules.

## Increase accuracy and resolve cross-reactivity

•*One of the most important implications of molecular-based allergy (MA) diagnosis is its ability to distinguish genuine sensitization from sensitization due to cross-reactivity.*

•*This information helps the clinician to determine whether a single, a few closely related, or several widely different allergen sources need to be considered.*

Allergic individuals may produce IgE antibodies to allergens that are either unique to a single species or common to many. Thus, the individual may show a genuine sensitivity to a given allergen or may show sensitivity to many unrelated species as a consequence of immunological cross-reactivity to structurally related allergens. In general, the closer the taxonomical relationship between species, the higher the degree of structural and immunological similarity between the allergens.

However, proteins with important biological functions are often well preserved and ubiquitous throughout related and unrelated species. Proteins are classified into protein families according to their biological function and structure [7]. Proteins within the same family share common epitopes and the same IgE antibody can bind to similar structures present in allergens from different allergen sources. These cross-reactive allergens give valuable information on potential sensitization and clinical reactions to several different sources. For example, IgE antibodies formed against either pathogenesis-related (PR)-10 protein family members Bet v 1, a birch pollen allergen, or Mal d 1, an apple allergen, cross-react and give rise to sensitivity to both birch and apple. Examples of other common cross-reacting allergen families are listed in Additional file 1: Table S1 [8,18-25]. It is worth noting that some cross-reacting molecules can cause clinically relevant symptoms, while others usually do not. While, MA studies have not fully elucidated the underlying mechanism governing cross-reactivity and symptom presentation, it is likely that analysis of the epitopes of key allergens will provide insight into this issue [26]. Currently, a large number of purified or recombinant allergens are commercially available. Additional file 2: Table S2 provides an overview of available allergens for *in vitro* testing, as of January 2013.

In contrast, specific allergens are markers for their respective allergen sources, allowing identification of the primary sensitizer. One of the most important clinical uses of MA is its ability to identify the offending allergenic molecule and to distinguish specific molecules from markers of cross-reactivity. Thus, the probability of a clinical reaction on exposure to different allergen sources may be defined, in some cases, by the pattern of sensitization to different allergens.

In the field of pollen-related food allergy, MA has demonstrated its ability to play an important role by increasing the accuracy of diagnosis. As an example of this, in peanut allergic patients, sensitization to Ara h 2 is considered a genuine marker for peanut that may induce systemic reactions, while Ara h 8 is a marker for cross-reactivity among food allergens and *Fagales* tree pollen and is mainly associated with mild, oral reactions [8,27]. Therefore, measuring IgE responses to certain food allergens may reduce the need for food challenges [28-30]. In patients sensitized to different pollen species, MA diagnostics is able to improve the resolution of a conventional diagnostics obtained by skin tests in a substantial number of cases, either by detecting new relevant sensitizations or by ruling out clinically irrelevant sensitizations caused by non-symptomatic cross-reactive allergens [11,31]. For example, MA diagnostics can help to distinguish baker’s asthma from pollen or wheat allergy [32].

In conventional SPTs, some allergens can be poorly represented in extracts because of the biological variability of the allergen source. For instance, Can f 5, a prostate-derived allergen produced by male dogs, is a dog allergen responsible for sensitivity in up to 38% of dog-allergic patients [33]. Allergen extracts used in skin tests, however, typically use dog hair as an allergen source. As a result, these skin tests routinely fail to identify patients’ sensitivity to Can f 5, likely due to its low concentration in dog hair [33]. Determination of the IgE response to Can f 5 using MA diagnostics may enhance the accuracy of dog allergy diagnosis.

When testing a limited panel of molecules, only what is measured can be detected; in other words, when using *Phleum pratense* positive SPT or sIgE test, Phl p 1 and Phl p5 will be able to define genuine sensitization, while Phl p 7 and Phl p 12 will identify sIgE for polcalcins and profilins, respectively. The presence of other molecules such as Phl p 2 and Phl p 4 could improve the accuracy of the diagnosis. If all of these molecules are studied, a fairly representative IgE profile for *P. pratense* would be obtained; if only one or a few molecules are evaluated, the characterization of the IgE profile would be less accurate. Thus, the descriptive quality of the sIgE profile will be based on the choice of tests prescribed by the clinician.

Nevertheless, it must be kept in mind that all allergy diagnostics, including MA, should be evaluated within the framework of a patient’s clinical history, because IgE sensitization towards a given allergen does not necessarily imply clinical responsiveness. This is of particular importance, since allergic patients respond in an individualized manner to exposure to allergens from various sources, i.e., every individual produces their own unique IgE antibody profile at the molecular level [12].

## Assess the risk and type of reaction

•*Molecular-based allergy (MA) diagnostics have emerged into routine care due to its ability to improve risk assessment, particularly for food allergies.*

•*Different foods contain unique allergenic molecules that are stable or labile to heat and digestion. The stability of a molecule and a patient’s clinical history help the clinician evaluate the risk of systemic versus local reactions. Labile allergens are linked to local reactions (typically oral symptoms) and cooked food is often tolerated, whereas stable allergens tend to be associated with systemic reactions in addition to local reactions.*

•*MA diagnostics may decrease the need for provocation testing and improve recommendations for allergen avoidance.*

Risk assessment of allergic individuals is one potential application of MA diagnostics. Since patient sensitization profiles may differ with regard to disease expression and severity, detecting “low-risk” versus “high-risk” molecules is an area of major interest that could reduce the use of potentially harmful diagnostic procedures such as challenge tests. Such knowledge may also improve allergy management recommendations to patients (e.g., exposure reduction). This has been shown with the use of MA diagnostics in food, venom, respiratory and latex allergy [8,34]. In addition, the sensitization profile of a patient may impact overall symptomatology, as poly-sensitization to several different allergens from a single allergen source may increase symptom severity [27,35].

Nevertheless, it must be noted that information may only be applicable to the specific population which has been studied, since it is known that both food and inhalant sensitization profiles and disease expression differ according to local exposures patterns characteristic of the geographical region [36].

### Food allergens

Generally, allergens resistant to heat and digestion often trigger more severe allergic reactions (i.e., anaphylaxis) compared to labile allergens, the latter which typically induce local symptoms such as oral allergy syndrome (OAS) (Table 1). In addition, the amount of a molecule present in a food source is also a parameter to take into consideration. The following text provides a few examples of how IgE sensitization to different allergens from a food allergen source can result in clinically unique reactions.

**Table 1:** **High**- **versus low**-**risk molecules from foods giving rise to anaphylaxis**

**Source**	**High risk**	**Low risk**
Peanut	Ara h 1, 2, 3, 9	Ara h 8, profilin, CCD
Hazelnut	Cor a 8, 9, 14	Profilin, CCD
Walnut	Jug r 1, 2, 3	Profilin, CCD
Soy	Gly m 5, 6, (4)	Profilin, CCD
Rosacea fruits	Pru p 3, Mal d 3	Pru p 1, Mal d 1, profilin, CCD
Wheat	Tri a 14, Tri a 19	Profilin, CCD

KEY: *CCD* = Cross-reactive Carbohydrate Determinant.

#### Peanut

Allergen sensitization profiles in peanut-allergic individuals have been extensively studied. IgE antibodies against storage proteins such as Ara h 1, 2, and 3 have been associated with genuine peanut reactions; in contrast, isolated sensitization to Ara h 8 (PR-10 protein and Bet v 1- homologue) is a marker of milder or local symptoms [24,27,35,37]. In southern Europe, the LTP (Ara h 9) is a prevalent sensitizing allergen that may act as a marker of severity, as it is associated with systemic and more severe reactions [8]. Further studies in other geographical regions are needed for Ara h 9 [36]. Finally, patients with profilin or CCD sensitization to peanut alone usually react with no or local oral symptoms, and heated peanuts may be tolerated.

#### Soy

Sensitization to Gly m 5 and/or Gly m 6 has been associated with severe reactions in allergic patients, while Gly m 4 (PR-10) is commonly associated with OAS [38]. Nevertheless, in birch pollen-allergic individuals, the combination of Gly m 4 sensitivity and intake of large amounts of midly processed soy, such as soy drinks, can induce a severe reaction [39]. Patients with profilin or CCD sensitization to soy alone usually exhibit no, or local oral symptoms, and heated soy may be tolerated.

#### Hazelnut

While sensitization to Cor a 1 (PR-10) is associated with local reactions like OAS, Cor a 8 (LTP) and storage proteins (e.g. Cor a 9 and Cor a 14) are more frequently recognized by IgE antibodies from patients with severe symptoms [40-42]. Patients with profilin (Cor a 2) or CCD sensitization to hazelnut alone usually exhibit no or local oral symptoms and heated hazelnuts may be tolerated.

#### Walnut

Severe reactions in walnut-allergic patients are associated with storage protein (Jug r 1, Jug r 2) or LTP (Jug r 3) sensitization [43]. Walnut allergens have not been available on the market until recently, as is reflected by the lack of recent clinical studies. Patients with profilin or CCD sensitization to walnut alone usually exhibit no, or local oral, symptoms and heated walnut may be tolerated.

#### Wheat

Sensitization to ω-5-gliadin (Tri a 19) is a risk factor for immediate allergic reactions in children and for systemic exercise-induced reactions in adults [44-46]. The wheat LTP (Tri a 14) shows some degree of cross-reactivity with other food LTPs, however more knowledge is needed about its prevalence and clinical implication. Patients with profilin or CCD sensitization to wheat alone usually exhibit no, or local oral, symptoms and heated wheat may be tolerated.

#### Rosaceae fruits

Apple, peach, and other stone fruits are members of the *Rosaceae* family. In patients allergic to these fruits, particularly to allergens such as PR-10 proteins (Mal d 1, Pru p 1) or profilins (Pru p 4), local, oral reactions are more frequent, since these protein families are sensitive to heat and digestion. In contrast, sensitization to LTP (Pru p 3), typical of the Mediterranean area, is associated with a wide range of clinical expressions (from asymptomatic to anaphylaxis), and is generally considered a risk marker for severe reactions including co-factor (e.g., exercise, alcohol or drugs) dependent anaphylaxis [25,47-50].

#### Egg

High levels of sIgE antibodies to ovomucoid (Gal d 1) have been identified as a risk factor for persistent egg allergy, including reactions to cooked/heated egg, while undetectable levels indicate tolerance to cooked egg [51].

#### Milk

Casein (Bos d 8) and beta-lactoglobulin (Bos d 5) sIgE antibodies are markers of persistent allergy to milk, including heated milk, in milk allergic patients while undetectable levels indicate tolerance to baked milk products [52].

#### Fish

Parvalbumins (e.g., Gad c1 and Cyp c 1) are the major allergens in fish and are typically stable to heat and digestion. Parvalbumins show a high degree of cross-reactivity whereby patients sensitized to one parvalbumin may also react to parvalbumins from other fish, including carp, cod, herring, plaice, mackerel, tuna, salmon, perch, and eel [53-55].

#### Shellfish

Allergic reactions to crustaceans may be caused by tropomyosin, which shows high degree of cross-reactivity across a wide variety of species, including mites [56]. Shrimp and other shellfish also contain other clinically relevant allergens, like sarcoplasmic calcium-binding protein and arginine kinases [57].

#### Meat allergy

Galactose-α-1,3-galactose (α-Gal) is a sugar structure found on glycoproteins and glycolipids of non-primate mammals and new world monkeys, but not on humans. IgE-antibodies specific for α-Gal (anti-α-Gal-IgE) may be associated with severe allergic symptoms and with delayed-type anaphylaxis [58,59]. α-Gal is also present on cat IgA which does not show high allergenic activity [60], and on gelatine containing material. It is assumed that sensitization to α-Gal can be induced by tick bites or certain parasite infections [61-63]. Clinically, α-Gal–sensitized patients may experience delayed immediate type reactions to red meat (beef, pork, goat, deer) anaphylaxis [58,59].

α-Gal is also present on the chimeric antibody cetuximab (cancer drug), and patients sensitized to α-Gal may react with anaphylactic reaction after the administration of cetuximab. Testing for α-Gal before administration of cetuximab should therefore be considered [64].

Bovine serum albumin (e.g. Bos d 6) is a heat labile allergen present both in milk and beef, which may cause cross-reactivity between different mammalian meat [65].

### Inhalants

#### Pet dander

Higher levels of sIgE antibodies against Fel d 1 are associated with asthma in cat-allergic individuals [66]. Recognition of more than three animal-derived allergens such as lipocalins (Mus m 1, Equ c 1, Fel d 4, Can f 1, 2), kallikrein (Can f 5), and secretoglobin (Fel d 1) has been associated with severe asthma in Swedish children [67]. More knowledge is needed in the area of pet allergy where many of the patients are poly-sensitized to several pets and the clinical history is often inconclusive, in addition the cross-reactivities between e.g. cat, dog and horse is not fully clarified at the MA level.

#### Pollen

Research in pollen allergy has focused on distinguishing genuine allergens from those that are cross-reactive, however, little is known regarding specific markers of severe reactions. Nevertheless, some sensitivities to specific allergens may be markers of more severe symptoms in pollen allergy, increasing the risk of systemic reactions during immunotherapy, such as Ole e 9 and the pollen LTP Ole e 7 [68].

Profilin sensitization is common among pollen allergic patients and it is usually associated with mild or no clinical symptoms. However, for a minority of patients, profilin may be a risk factor for more severe reactions in olive pollen-allergic individuals and in patients allergic to certain plant foods like melon or citrus [8,25].

#### Mites

Although no specific sensitization profile has been described as a risk factor for lower airway disease or disease severity, a higher sIgE/IgG4 ratio for Der p 2 has been associated with asthma [69,70]. Der p 10 (tropomyosin) is a minor allergen in mite-allergic patients, however it may still indicate a risk for allergic reactions to shellfish or snail, which can be severe [71].

#### Molds

In hypersensitivity reactions to *Aspergillus fumigatus*, the presence of IgE antibody reactivity to Asp f 2, 4, and 6 may suggest allergic bronchopulmonary aspergillosis (ABPA) [72], whereas sensitization to Asp f 1 and/or Asp f 3 may be more indicative of allergic asthma [73]. These associations must still be confirmed in other patient populations.

#### Cockroach

It was recently described that sensitization to Per a 2 correlates with severity of airway allergy in cockroach-allergic patients [74]. Per a 2 is currently not commercially available for *in vitro* testing; however, the Per a 2 homologue Bla g 2 is available. Cockroaches also contain cross-reactive tropomyosin (Bla g 7), which indicates a risk for allergic reactions to shellfish or snail, which can be severe [71].

### Other allergens

#### Latex

Sensitization to Hev b 8 (profilin) seems to be clinically irrelevant and not related to clinical latex reactions. The other latex allergens are linked to clinical reactions; however, no association between allergens and severity of reactions has been identified so far [75,76]. The cross-reactive allergen responsible for the so called latex-fruit syndrome are not fully clarified, although data indicate that Hev b 5, 6 and 11 play a role [8,77].

#### Hymenoptera venoms

Most hymenoptera venom allergens possess CCDs that are responsible for a portion of clinically irrelevant IgE antibody cross-reactivity between bee and wasp venom. Detection of recombinant venom allergens can discriminate between genuine venom sensitization and cross-reactivity due to CCDs in patients with double-positive IgE results from traditional venom tests that are based on allergen extract [8,13,14].

## Specific immunotherapy

•*Molecular-based allergy (MA) diagnostics represents a useful tool to distinguish genuine sensitisations from cross-reactions in poly-sensitized patients, when traditional diagnostic tests and clinical history are unable to identify the relevant allergen(s) for specific immunotherapy (SIT).*

•*Given that SIT is an expensive treatment typically used over longer periods of time (3 to 5 years), correct diagnosis, selection of truly eligible patients, and identification of primary sensitizing allergen(s) are important for optimal and cost-effective patient management.*

Specific immunotherapy (SIT) involves the administration, either subcutaneously or sublingually, of an extract of the allergen responsible for clinical symptoms to induce tolerance and reduce reactivity (i.e., symptoms) to the allergen itself [78,79]. This is achieved through complex immune modifications that involve both humoral and cell-mediated immunity [80]. As a paradigm, allergen immunotherapy is “specific”, meaning that it only modifies the immune response against the allergen for which the vaccination is being performed. As a consequence, a precise etiological diagnosis is required for the prescription of SIT, whereby the allergen responsible for clinical symptoms must be unequivocally identified. In some patients, a detailed clinical history and traditional extract-based IgE testing (SPT and/or *in vitro* sIgE) is sufficient to identify the relevant allergen(s) [81]. This is especially true in the case of allergy to plants with a well-defined pollen season, which does not overlap significantly with that of other plants or other allergen sources.

However, the complexity of diagnosis increases when the patient demonstrates poly-sensitization by traditional diagnostic tests based on allergen extracts and their clinical history is not sufficient to clarify the nature of the sensitization. This may occur in a relatively high proportion of patients [82,83]. In the United States, for instance, such cases would involve preparing a vaccine for SIT by mixing together all of the allergens that a patient tests positive for [84,85]. Mixing numerous allergens appears to achieve good clinical efficacy; however, there may be an inability to identify the responsible allergen in the case of adverse events [86].

It is well recognized that, in many cases, multiple positive results obtained with allergen extracts (i.e., SPT and/or *in vitro* sIgE) are due to the presence of cross-reactive allergens in the diagnostic extracts [87,88]. Certain proteins (e.g., profilins, polcalcins, LTPs, PR10, tropomyosins) are highly conserved in a wide variety of species. For instance, a patient who is primarily sensitized to grasses may also test positive for birch with SPT [89]. This cross-reactivity occurs because the birch extract used in SPT contains profilin (e.g., Bet v 2), which are largely similar to those in grasses (e.g., Phl p 12). Indeed, the use of recombinant/purified allergens would allow for the discrimination between genuine sensitizations and cross*-*reactivities. In the example mentioned above a patient with sIgE antibodies against Phl p 1 and Phl p 5 but no sIgE to Bet v 1 is truly sensitized to grass. If sIgE antibodies to Phl p 12 (profilin) were also detected, profilin sensitization would probably be responsible for the positive SPT result obtained with birch extract, which contains profilin as well. Thus, using knowledge gained through the identification of allergens, SIT would be prescribed for grass only. Similarly, if a patient is sensitized to a traditional house dust mite extract, but their IgE antibodies are specifically directed against Der p 10 (tropomyosin) and not to Der p 1, 2/ Der f 1, 2, SIT for mites should not be given, because mite extracts mainly contain Der p 1, 2/Der f 1, 2 and have variable or low amounts of Der p 10. Molecular diagnostics can also improve the selection of patients for hymenoptera venom SIT. Sensitization to the major allergens Api m 1 of honeybee and Ves v 5 and/or Ves v 1 of yellow jackets may be helpful in discriminating between true double bee and wasp sensitization and cross-reactivity due to CCDs [13].

In addition, most commercial allergen extracts used in SIT are well standardized for major allergens, but contain only minimal or variable amounts of minor allergens [90,91]. Thus, patients with sensitization to minor allergens alone will likely not receive sufficient amounts of allergen to achieve a successful outcome by SIT. A recent study reported that patients receiving a 2-year course of SIT with either birch or grass pollen had a much more favourable outcome with SIT when sensitization to the marker allergens of birch or grass pollen were detected compared to patients sensitized to only minor, cross-reactive allergens [92].

In poly-sensitized patients, the most relevant sensitizing allergens for which SIT should be prescribed can be more clearly identified with MA diagnostics. A recent study reported that the use of MA diagnostics modified the prescription of SIT compared to SPT in more than 50% of patients [11], suggesting that poly-sensitized patients are at risk of incorrect SIT prescription.

Theoretically, a detailed identification of molecules to which IgE antibodies are directed against would allow for tailored SIT based only on allergens with a documented IgE response for each patient. In practice, this does not seem feasible. First, the number of possible combination of sensitization profiles is large when taking into consideration all allergenic sources, [12]; second, recombinant vaccines do not perform better than traditional allergen extracts, as observed in some studies [93,94]; and third, each single recombinant/purified allergen would need to be individually tested and registered, which carries a substantial financial burden for manufacturers. Thus, the reality of patient-tailored SIT is still a distant prospect [95].

## Micro-array technology

•*Molecular multiplex platforms help the clinician to obtain an overview of the sensitization profile of the patient with a small amount of serum and to identify cross-reacting, unanticipated, or potentially high risk allergens.*

•*Currently one multiplex platform is available on the market (the Immuno-Solid phase Allergen Chip (ISAC) platform). While not interchangeable, ISAC results are similar with those obtained from singleplex platforms. However, at low sIgE levels ImmunoCAP is more sensitive than ISAC and this should be considered when interpreting ISAC results with regard to patient clinical history.*

•*Poly-sensitized pediatric and adult patients in whom sensitization to cross-reacting allergens is suspected are most suited for ISAC testing, especially when both food and airborne allergens are involved.*

MA diagnostics has been available on singleplex platforms such as the ImmunoCAP, ImmuLite, and HyTech platforms for many years. These platforms use panels of single allergens together with the corresponding allergen extract. Presently, MA diagnostics can also be performed using multiplex technology to measure sIgE antibodies against multiple allergens in a single assay [96]. This technique allows for the testing of a large number of allergens using a small amount of serum. Several research platforms have been described in the literature, whereof one, ImmunoCAP Immuno-Solid Phase Allergen Chip (ISAC) (Phadia AB) is commercially available. The first European Conformity (CE)-certified version of ISAC was developed and launched by VBC-Genomics, Vienna, in 2003. The initial chip contained 23 allergens, and, since then, it has continually been improved by providing a larger number of allergens. In 2007, the chip contained 103 allergens and in 2011 the ISAC 112 chip was made commercially available.

ImmunoCAP ISAC is a miniaturized immunoassay platform, where allergens are immobilized in a microarray. A minimum of 30 μl of serum or plasma, obtained from either capillary or venous blood, is needed to probe the chip. The assay consists of a polymer-coated glass slide that contains four microarrays, suitable for assaying four simultaneous samples. The allergens are spotted in triplicate and covalently immobilized on the chip. The procedure consists of the following two main steps: (1) IgE antibodies from a patient’s sample bind to the immobilized allergens and (2) allergen-bound IgE antibodies are detected by a fluorescence-labeled anti-IgE antibody. The test procedure, including all washing and incubation steps, can be performed in a total assay time of less than four hours. Fluorescence is measured with a laser scanner and results are evaluated using a Microarray Image Analysis (MIA) software, which provides an automatic readout of the results. In addition, add-on software is available (ISAC Xplain) that delivers evidence-based allergen information relevant for the individual patient. Using a standard calibration curve, results are reported within a range of 0.3 to 100 ISAC Standardized Units (ISU-E), giving a semi-quantitative indication of IgE antibody levels. This differs from the units used to report ImmunoCAP results (kU/L), and as such, these measurements are not interchangeable, although they correlate well [97]. Furthermore, it must be born in mind that the ImmunoCAP technology measures IgE binding under conditions of excess of immobilized allergen whereas ISAC uses low amounts of immobilized allergen allowing for competition with allergen-specific isotypes other than IgE.

Several studies, summarized in Table 2, have analyzed the reproducibility of this immunoassay and have compared the ISAC chip with other methods of measuring sIgE [31,97-102]. Overall, the results of the ISAC assay are reproducible at a level that is generally accepted and agreed upon. However, special attention is recommended when samples contain low levels of sIgE (0.3–1 ISU-E), as a higher degree of variability in low-level results has been observed. When comparing ISAC with other sIgE measuring assays such as the singleplex ImmunoCAP platform, concordance of results vary between allergens tested [31,76,97-99,102,103]. Comparative data with the ImmuLite or HyTech platforms are not available in the literature. Nevertheless, newer versions of ISAC have resolved a number of observed discrepancies. Because there is a higher degree of between assay variability for ISAC compared to ImmunoCAP, ISAC is generally not recommended for monitoring quantitative IgE levels over time in clinical routine. While no interference from very high total IgE has been observed [98], a potential for interference between IgE and other isotypes, principally IgG, has been indicated (e.g., during SIT) [104].

**Table 2:** Studies comparing different techniques for specific IgE determinations

**Techniques compared**	**Allergens**	**Main findings**	**References**
ImmunoCAP & ISAC 50	HDM, cat dander, birch, grass, and mugwort pollen	ROC curves demonstrated that CAP and ISAC performed equally well in cat, birch, and grass pollen. ISAC was slightly less sensitive in HDM and displayed a reduced sensitivity in mugwort pollen.	Wöhrl et al. [99]
ImmunoCAP & ISAC prototype	Betula and grass allergens	Comparable sensitivity between CAP and ISAC.	Jahn-Schmid et al. [100]
ImmunoCAP & ISAC 103	grass and cypress pollen	Showed similar diagnostic performance.	Cabrera-Freitag et al. [101]
ImmunoCAP & ISAC 103	Multiples allergens	Concordance was 78.65% for positive results.Concordance was 93.57% for negative results.	Gadisseur et al. JACI [98]
Reproducibility of ISAC 103	rApi g 1, rBet v 2, nBos d 4, nGal d 1, nGal d 2, nGal d 3, rHev b 8, rPhl p 5, rPhl p 6, and rPhl p 7	Excellent intra-slide, intra-assay, and inter-assay variability. rApi g 1, nGal d 3, and rPhl p 6 showed high variability in the individual analyses.	Cabrera-Freitag et al. [101]
ImmunoCAP & ISAC 103	Latex allergens	Similar performance	Ebo et al. [76]
ImmunoCAP, ISAC 103, & ADVIA-CENTAUR	Pollen allergens	The 3 diagnostic methods were in agreement in 62.5% of cases. ISAC showed a deficiency in the detection of sensitivities to *Salsola* and *Plantago*; Advia-Centaur did not detect sensitizations to cypress. The concentration of sIgE in ISAC and ADVIA were significantly correlated for most pollen allergens.	Lizaso et al. [31]
ImmunoCAP & ISAC 103	103 ISAC molecules	For low ISU values (0.3 to 1), the within-assay CV was very high (>100%), as expected; for medium (1 to <15) and high (15 or higher) ISU values, the CV was 17% and 8% respectively. The corresponding between-assay CVs were >100%, 33%, and 13.2%, respectively.	Melioli et al. [97]
ImmunoCAP & ISAC 103	Alt a 1	Similar performance	Twaroch et al. [103]

**KEY**: *HDM*: House Dust Mite; *ISAC*: Immuno-Solid phase Allergen Chip; *ISU* = ISAC Standard Units; *ROC*: Receiver-Operating Characteristic curve; *sIgE*: Specific Immunoglobulin E.

The use of allergen microarrays has not only improved allergy diagnosis [15,98] and optimized management of SIT [11], some studies also indicate that microarrays can be used to analyse the allergic march [105], sensitization in preclinical stages and molecular spreading [106,107]. Although sensitivity compared to ImmunoCAP is still often lower, ISAC can have high clinical relevance by detecting sensitization patterns to important allergens and cross-reacting groups. In addition, the broad allergen panel offers the potential for identifying unanticipated triggers. In a recent study that followed patients for over 30 years, sensitization detected specifically to Aln g 1 led to the identification of a newly planted and imported alder tree (*A. spaethii*) as the most probable source responsible for a number of cases of unexplained hay fever symptoms that were present earlier in the year than expected. This was supported by the fact that these hybrid alder trees flowered earlier than allergen sources native to the area [108].

Table 3 summarizes the advantages and limitations of different IgE measurement techniques. When deciding if and when to use the microarray technology, it is helpful to consider the number of allergens to be tested; in general (depending on the local price and reimbursement system), if more than 10 to 12 allergens are required for an accurate diagnosis using singleplex tests, then a microarray test may be preferable both for the information obtained and for economic reasons [15].

**Table 3:** Advantages and disadvantages of ISAC, immunoCAP, and skin prick tests

	**Advantages**	**Disadvantages**
ISAC	• 30 μl of serum or plasma (capillary or venous blood)	• Manual method
	• 112 allergens can be assayed in parallel	• Semi-quantitative assay
	• Natural and recombinants proteins	• Less sensitive
	• Less allergen needed (approximately 100, 000-fold, pg vs. μg) per assay	• More variability in the inter-assay analysis for certain allergens
	• No interference from very high total IgE	• Greater coefficient of variation
		• Some allergen sources are not included
		• Less appropriate for monitoring sensitization
		• Potential interference between IgE and other isotypes, principally IgG
ImmunoCAP	• Automatic method	• 40 μl of serum per allergen
	• Quantitative assay	• One allergen per assay
	• High sensitivity	• Detect low-affinity antibody that may have little to no clinical relevance
	• Lower coefficient of variation	
	• Natural or recombinants proteins or crude extracts	
	• Appropriate for monitoring sensitization	
Skin prick test	• High sensitivity (extract-dependent)	• Manual
	• Immediate reading	• One allergen per prick
		• Only crude extracts
		• Not appropriate for monitoring sensitization

**Figure 1:**
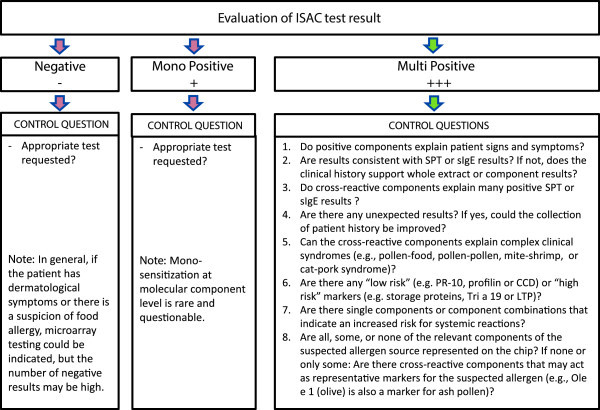
ISAC Interpretation flow chart.

The interpretation of the results of a 112-allergen assay may be challenging, even for the experienced and trained ISAC user. First, the clinical relevance of the different allergens must be considered. Second, the results must be evaluated in relation to traditional diagnostic tests. Finally, and most importantly, the results must be evaluated with regard to the patient’s clinical history. Indeed, while the vast majority of molecules cover the spectrum of positive traditional tests, it is known that ISAC results for some allergen sources such as cashew nut, sesame, dog, mugwort, and ragweed can be negative, even when the extract-based test is positive. This is obviously the case if the triggering allergen is not present on the chip. The standard strategy used to evaluate an ISAC result is outlined in Figure 1.

In summary, while not interchangeable, the results generated with the ISAC chip are similar to those obtained with the ImmunoCAP platform. One disadvantage to the technique is that the sensitivity of ISAC is lower than that of ImmunoCAP, particularly when sIgE levels are low. However, the ability to use a small amount of serum to obtain a patient’s sensitization profile, identify cross-reacting allergens, and detect unsuspected or potentially harmful allergens are advantages of the use of ISAC in allergy diagnosis in patients with allergy-like symptoms (e.g. asthma, rhinitis, eczema, urticaria idiopathic anaphylaxis or eosinophilic esophagitis (EoE)).

## Patients most likely to benefit from molecular-based allergy diagnostics

•*Molecular-based allergy (MA) diagnosis is most useful for selection of SIT, evaluation of cross-reactivity, and assessment of severity of reaction associated with various allergens.*

•*Patients who are poly-sensitized, have an unclear symptom and/or sensitization pattern, or who do not respond to their treatment may be routinely evaluated using MA diagnostics when available.*

•*Mono-sensitized patients with a clear case history and symptom profile may not benefit from MA diagnostics compared to traditional diagnostic tests.*

MA diagnostics offers several advantages useful for the examination of allergic patients with symptoms like e.g., asthma, rhinitis, eczema, urticaria, gastrointestinal, oral allergy syndrome or anaphylaxis. Identification of genuine sensitization is as important as the identification of secondary sensitizations caused by cross-reacting allergens.

MA diagnostics, based on either the physician’s choice of single allergens or the use of a microarray, offers a large amount of information pertaining to the IgE profile of sensitized patients. This information is mainly useful for three purposes. First, MA diagnostics is helpful in the identification of a genuine sensitization to an allergen source, particularly when SIT is being considered. MA diagnostics is often essential to the accuracy of prescribed SIT for a large proportion of allergic patients [8]. Second, MA diagnostics can detect sensitization to certain cross-reacting protein families of allergens, thereby contributing to identify the triggering allergen source and to improve the recommendations made to patients regarding exposure avoidance. Finally, MA diagnostics helps to assess the risk associated with certain allergens (i.e., type of reaction, local or systemic). For example, sensitization to LTP or storage proteins may cause severe, systemic reactions in allergic patients while profilin, CCD and PR-10 proteins generally are associated with mild, local reactions in food allergy.

From among those potentially eligible for MA diagnostics, different patient categories can be defined. In most patients, MA diagnostics may be considered a useful and interesting, but not essential, tool, particularly when only symptomatic treatment is prescribed. Mono-sensitized patients (e.g., to pet or mite allergens) and patients with a clear case history and symptom profile do not generally seem to derive benefit from MA diagnostics compared to traditional diagnostic tests.

Previously, patients who were sensitized to one or two allergen sources were the most prevalent patient type in clinical practice; currently, they are becoming a minority, mainly in developed countries. In fact, poly-sensitized pediatric and adult patients with complex symptoms, as well as patients in whom sensitization to cross-reacting allergens is suspected, should be carefully considered for MA evaluation. Within this population, patients with documented poly-sensitization to one or more inhalants, but also suffering from food allergy (i.e., from less severe manifestations such as OAS to more severe, including anaphylaxis, asthma or eczema) should be routinely considered for evaluation using MA diagnostics. In addition, MA diagnostics may offer additional information for early diagnosis of allergies and may aid in the monitoring of the evolution of the allergic disease, useful for preventive indications to the patient.

In conclusion, current guidelines of allergy diagnosis should recommend a thorough clinical investigation as a first-line approach, followed by allergen extract testing using *in vitro* sIgE or SPT tests as a second-line approach, and as a third step MA diagnostics. For experienced users MA may be included in second-line testing.

## Unmet needs

•*Molecular-based allergy (MA) diagnostics enhances the clinical utility of specific IgE (sIgE) antibody-based allergy diagnostics nevertheless; a number of unmet needs have yet to be addressed.*

Molecular analysis of allergen sensitization patterns can enhance the clinical utility of allergy tests based on extracts. In selected cases it may also reduce the need for challenge testing for food allergies and may also improve the selection of SIT prescription. However, there are a number of unmet needs pertaining to MA diagnostics:

1) Large-scale, population-based multicenter studies are needed to further define in which categories of patients MA diagnostics may be beneficial.

2) The practical use and selection of allergens in MA diagnostics need to be evaluated in large studies that include well-characterized patients and healthy, sensitized controls representative of different geographical regions.

3) Evaluation of the incremental benefits relative to the incremental costs for MA diagnostics, by way of cost-utility studies, is needed. These studies should compare the effectiveness of MA diagnostics with the traditional *in vitro* sIgE or SPT techniques that are currently available.

4) Identification and clinical evaluation of the most relevant allergens have to be further investigated in many allergen sources.(e.g., nuts, molds, tree and weed pollen).

5) Training efforts in both the clinical and research settings is warranted, with a focus on developing this new “molecular” era in allergology.

6) Development of clinical decision support is needed to prevent misinterpretation and improve knowledge as the amount of information obtained from MA diagnostics may be complex, especially as the evidence for MA is rapidly progressing.

There are additional needs in the field of allergy diagnostics including traditional tests based on extracts. Currently there is one published cost effectiveness analysis on food allergy diagnostics [109]. In this guideline document, economic evidence shows that both IgE antibody testing and skin prick testing are cost effective compared to clinical anamneses without testing. Since MA diagnostics increase the accuracy in selected food allergies (e.g. peanut allergy) and selection of SIT prescription compared to traditional tests based on extracts, the cost effectiveness should logically increase for these particular scenarios. However, the fact that only one cost effectiveness analysis in food allergy is available underlines the need for more cost effectiveness analysis in allergy diagnostics.

There is also a need for characterization and standardization of allergen concentrations in allergen extracts that are used in diagnostic testing and treatment.

## Summary and conclusions

•*International guidelines recommend a thorough clinical case history as a first-line approach and allergen extract-based IgE tests (in vitrospecific IgE or skin prick test) as a second-line investigation.*

•*Molecular-based allergy (MA) diagnostics is considered a third-line approach to be used for patients in whom first- and second-line investigations were inconclusive. For experienced users MA may be included in second-line testing.*

•*MA diagnosis is a new and complex procedure that, in the near future, will represent a standard tool in the allergist’s armamentarium. Educational programs on MA diagnostics for allergists are needed.*

MA diagnostics was developed more than a decade ago. The recent availability of a greater number of allergens has substantially modified the diagnostic approach used by many allergists. Currently, international guidelines recommend a thorough clinical case history as a first-line approach and allergen extract-based IgE tests (*in vitro* specific IgE or skin prick test) as a second-line investigation for the identification of the allergen source responsible for a patient’s symptoms. SPT and *in vitro* sIgE tests provide similar information and the associated advantages and disadvantages of both types of tests are dependent on the clinical case. For the majority of patients, first- and second-line investigation is sufficient to define the nature of a patient’s allergy. Molecular-based allergy (MA) diagnostics is considered a third-line approach to be used for select patients in whom first- and second-line investigations were inconclusive. For experienced users MA may be included in second-line testing.

Traditional diagnostic tests have been considered sufficient for the identification of the best SIT prescription in the majority of patients. With the identification of specific and cross-reacting allergens, a number of new diagnostic and therapeutic options are available to allergists, including the ability to choose the allergen composition for SIT.

MA diagnostics is relatively expensive compared with traditional tests, especially with regard to the microarray technology. Economic consideration or budget limitations may influence the decision in the individual patient, whether using a singleplex or multiplex approach. The number of allergens to be tested may influence this decision, both for economical reason, amount of information gained and for the overall serum volume required (especially in young children).

When making the choice to use the microarray diagnostics, it is important to consider the primary advantage which is that with a small serum or blood sample, a broad spectrum analysis of a patient’s IgE profile can be performed. However, a disadvantage is that patients may be at risk of revealing unanticipated sensitivities, possibly to potentially harmful molecules. Although this could also be considered as an advantage, the interpretation of such sensitization in clinically unresponsive patients is difficult or even impossible.

Although MA diagnostics is a complex area, it provides novel and relevant information for the allergist and will soon become a standard tool in the allergist’s armamentarium. Educational programs training allergists on the use and interpretation of MA are highly needed.

## Competing interests

G Walter Canonica: Consultant, speaker, or researcher with the following commercial companies: Thermo Fisher, Alk-Abello, Allergopharma, Allergy Therapeutics, Anallergo, Hal, Lofarma, Stallergenes.

Ignacio Ansotegui: Consultant and or speaker with the following commercial companies: Faes Farma, Bial-Arístegui, Pfizer and Sanofi.

Ruby Pawankar: None.

Peter Schmid-Grendelmeier: Consultant and or speaker for ThermoFisher, Phadia, Scientific AG, Siemens, Diagnostics AG, Buhlmann Labs AG.

Marianne van Hage: Member of the Clinical Advisory Board of Biomay, Vienna, Austria; Researcher in scientific trial in collaboration with Professor Valenta at the Medical University of Vienna.

Carlos E Baena-Cagnani: Consultant, speaker, or researcher with the following commercial companies: Novartis, Sanofi, Stallergenes, FAES, Lofarma.

Giovanni Melioli: Consultant, speaker, or researcher with the following commercial companies: Thermo Fisher - Phadia, Milano, italy Bruschettini srl, genova, Italy Lallemand Pharma, Lugano (CH).

Carlos Nunes: None.

Giovanni Passalacqua: Consultant, speaker, or researcher with the following commercial companies: Anallergo Almirall AstraZeneca GSK Lofarma Menarini MSD Phadia Stallergenes.

Lanny Rosenwasser: None.

Hugh Sampson: Allertein Therapeutics, LLC - consultant Danone Scientific Advisory Board - consultant DBV - unpaid consultant Novartis - unpaid consultant University of Nebraska - FAARP advisory board -consultant NIAID - NIH - research funds to institution FARE - research funds to institution.

Joaquin Sastre: Consultant, speaker, or researcher with the following commercial companies: Novartis, GSK, Stallergenes, ALK, Thermofisher, FAES, Mundipharma, MSD, FAES FARMA, Mundipharma, Roche, Gennetech, GSK, Novartis.

Jean Bousquet: None.

Torsten Zuberbier: Consultant, speaker, or researcher with the following commercial companies: AnseIl, Bayer Schering, DST, FAES, Fujisawa, HAL, Henkel, Kryolan, Leti, Merck, MSD, Novartis, Procter and Gamble, Sanofi-Aventis, Schering Plough, Stallergenes, UCB.

Katrina Allen: Consultant, speaker, or researcher with the following commercial companies: Abbott, Danone, Pfizer.

Ricardo Asero: Consultant, speaker, or researcher with the following commercial companies: Phadia/Thermo-Fisher Allergopharma Lofarma SpA ALK-Abellò Malesci Mediolanum Allergy Therapeutics.

Barbara Bohle: Consultant, speaker, or researcher with the following commercial companies: Biomay AG, Vienna, Austria Bencard Allergie GmbH, Vienna, Austria.

Linda Cox: None.

Frederic de Blay: None.

Motohiro Ebisawa: None.

René Maximiliano Gómez: None.

Sandra González-Díaz: Consultant, speaker, or researcher with the following commercial companies: GSK MSD Pfizer Novartis Allmiral.

Tari Haahtela: None.

Stephen Holgate: Consultant, speaker, or researcher with the following commercial companies: Synairgen, Novartis, MSD, Stallergenes, Crescendo Biologics, Sterna, Amgen, BI.

Thilo Jakob: Consulting with Thermo Fisher Scientific.

Mark Larché: Circassia Ltd. Consultant, stockholder Adiga Life Sciences: Consultant Adiga Life Sciences: Research contracts Sanofi USA: Consultant Air Canada: Consultant.

Paolo Maria Matricardi: Consultant, speaker, or researcher with the following commercial companies: Allergopharma, ALK, ThermoFisher Scientific.

John Oppenheimer: Consultant: GSK, AZ, Sunorium, Myelin Research: GSK, Novartis, BI, Meddimune Chairman of the Board: ABAI Associate Editor: Annals of Allergy Asthma and Immunology.

Lars K. Poulsen: I have occasionally been speaking at Thermo Fischer Scientifc Symposia. Part of our research in diagnosis has been sponsored by Siemens or Thermo Fischer Scientific.

Nelson Rosário: Consultant, speaker, or researcher with the following commercial companies: Danone, MSD, Novartis, Aché, Sanofi, Takeda,Nestlé.

Marc Rothenberg: None.

Mario Sánchez-Borges: Consulting with Novartis, Sanofi Aventis.

Enrico Scala: None.

Rudolf Valenta: Consultant for Phadia/Thermofisher, Uppsala, Sweden and BIOMAY AG, Vienna, Austria.

## Supplementary Material

Additional file 1: Table S1 Common cross-reacting protein families. Click here for file

Additional file 2: Table S2 Commercially available allergen molecules for *in vitro* sIgE testing from three providers (ThermoFisher, Siemens and Hycor). Click here for file
